# Role of CX_3_CR1 Receptor in Monocyte/Macrophage Driven Neovascularization

**DOI:** 10.1371/journal.pone.0057230

**Published:** 2013-02-21

**Authors:** Arun H. S. Kumar, Kenneth Martin, Elizebeth C. Turner, Chirlei K. Buneker, Karim Dorgham, Philippe Deterre, Noel M. Caplice

**Affiliations:** 1 Centre for Research in Vascular Biology (CRVB), Biosciences Institute, University College Cork, Cork, Ireland; 2 Laboratoire Immunité et Infection, UMR-S 945 INSERM-UPMC, Paris, France; New York Medical College, United States of America

## Abstract

Monocyte/Macrophages are implicated in initiation of angiogenesis, tissue/organ perfusion and atherosclerosis biology. We recently showed that chemokine receptor CX_3_CR1 is an essential regulator of monocyte/macrophage derived smooth muscle cell differentiation in the vessel wall after injury. Here we hypothesised the contribution of CX_3_CR1- CX_3_CL1 interaction to in vivo neovascularization and studied the functional consequences of genetic and pharmacologic targeting of CX_3_CR1 in formation, maturation and maintenance of microvascular integrity. Cells functionally deficient in CX_3_CR1 lacked matrix tunnelling and tubulation capacity in a 3D Matrigel assay. These morphogenic and cytokinetic responses were driven by CX_3_CL1-CX_3_CR1 interaction and totally abrogated by a Rho antagonist. To evaluate the role of CX_3_CR1 system in vivo, Matrigel plugs were implanted in competent CX_3_CR1^+/gfp^ and functionally deficient CX_3_CR1^gfp/gfp^ mice. Leaky microvessels (MV) were formed in the Matrigel implanted in CX_3_CR1^gfp/gfp^ but not in CX_3_CR1^+/gfp^ mice. In experimental plaque neovascularization immature MV phenotype was observed in CX_3_CR1^gfp/gfp^ mice, lacking CX_3_CR1 positive smooth muscle-like cells, extracellular collagen and basement membrane (BM) laminin compared to competent CX_3_CR1^+/gfp^ mice. This was associated with increased extravasation of platelets into the intima of CX_3_CR1^gfp/gfp^ but not functionally competent CX_3_CR1 mice. Pharmacologic targeting using CX_3_CR1 receptor antagonist in wild type mice resulted in formation of plaque MV with poor BM coverage and a leaky phenotype. Our data indicate a hitherto unrecognised role for functional CX_3_CR1 in Matrigel and experimental plaque neovascularization in vivo, which may buttress MV collectively in favour of a more stable non-leaky phenotype.

## Introduction

The monocyte–macrophage system (MPS) exhibits numerous functions with respect to vascular remodeling which include classical phagocytosis, regulation of antigen presentation and atherosclerotic plaque formation [1], [2], [3], [4]. MPS has also been implicated in paracrine-mediated pro-angiogenic and vascular structure changes orchestrated in response to cues as diverse as atherosclerotic plaque progression, tissue injury and ischemia, inflammation and tumour infiltration [5], [6], [7], [8], [9], [10], [11]. For instance, macrophages have been reported to tunnel through stromal architecture of numerous tissues including the heart and the retina connecting putative microvascular networks [4], . However, the mechanism and molecular regulators underlying this process are largely unknown and it remains unclear whether this is a generalized feature of MPS infiltration of tissues or if it represents a more specialized function of cellular components of MPS that might be a target for pharmacologic modification.

We have previously identified a CX_3_CR1 monocyte subpopulation capable of phagocytic functions typical of classical MPS as well as mural vascular smooth muscle cell-like functions that occur subsequent to CX_3_CL1-CX_3_CR1 interaction in the injured vessel wall [16], [17]. Interference with CX_3_CL1-CX_3_CR1 interaction was also observed to decrease neointima formation and atherosclerosis in murine animal models [17], [18], [19], thus implicating the CX_3_CL1-CX_3_CR1 axis in atherosclerotic plaque development. Moreover polymorphisms in CX_3_CR1 receptors have been associated with variability in prevalence of human atherosclerosis and coronary artery disease [19]. Given that monocyte/macrophages are proposed to participate in angiogenesis [1], [4], [20], [21], [22] we hypothesized that CX_3_CL1-CX_3_CR1 signaling may also be implicated in mural cell fate determination during plaque microvessel formation both in terms of cell recruitment to the perivascular space but also in cytoskeletal re-organization of recruited mural cells that integrate into nascent and maturing microvessels. In the current study we selected the chemokine receptor CX_3_CR1 as a transgenic marker allowing baseline and loss of function status to be examined in mice that had green fluorescence protein knocked in at one or both CX_3_CR1 alleles [17], [23]. This facilitated simultaneous in vivo tracking of a major monocyte/macrophage subset into evolving neovascularization networks in solid matrix and in medium sized vessel wall microvasculature. These transgenic models also facilitated interrogation of loss of CX_3_CR1 function effects on CX_3_CR1 cell recruitment to the perivascular space, mural cell integration into evolving microvasculature, microvessel maturation and extracellular matrix production and microvessel haemorrhage and permeability. In vitro studies evaluated the signaling pathway downstream of CX_3_CR1 activation and its effects on cytoskeletal re-organization and solid matrix tunneling of CX_3_CR1 positive cells.

We show here for the first time that interaction between CX_3_CL1 and CX_3_CR1 initiates signaling through a Rho dependent pathway that promotes cell tubulation and tunneling through extracellular matrix. Moreover, functional competence of CX_3_CR1 to interact with its cognate ligand CX_3_CL1 is essential for microvessel maturation and expansion, and monocyte/macrophage-derived mural cell integration into evolving vasculature during neovascularization in vivo. Finally, CX_3_CR1 deficiency or therapeutic targeting with a peptide-antagonist reduces experimental plaque and Matrigel neovascularization but favours a leaky, haemorrhagic microvessel phenotype.

## Materials and Methods

### Animals

All *in vivo* procedures were approved by University College Cork Animal Experimentation Ethics Committee. Mice (male) employed in experiments were 8–12 weeks of age. Initial C57BL/6J breeding colonies were from Charles River UK. Transgenic CX_3_CR1^gfp^ (C57BL6/J background) mice [17] [23], in which either one (CX_3_CR1^gfp/+^) or both (CX_3_CR1^gfp/gfp^) copies of the *CX_3_CR1* gene were interrupted by enhanced gene fluorescent protein (eGFP) and were originally from the European Mutant Mice Archive (EMMA).

### CX_3_CR1 peptide antagonist (F1)

The CX_3_CR1 peptide antagonist (F1) was engineered from phage library of CX_3_CL1 mutants consisting of modified N terminus (QHHGVT sequence of the native CX_3_CL1 replaced with ILDNGVS). F1 peptide was purified from E. Coli inclusion bodies using standard procedures and its specificity to CX_3_CR1 receptor and pharmacodynamics has previously been described [24]. Based on the static adhesion assay and prior pharmacodynamics data [24], 50 µg of F1 peptide was dissolved in 100 µl of saline and administered intraperitoneally every third day from the 2^nd^ to 4^th^ week post carotid artery ligation.

### Static adhesion of mice bone marrow cells to CX_3_CL1 coated plate

48-well plates were coated for 1 hour with a 1∶200 dilution of a mouse antipolyHis tag antibody (HIS-1, Sigma Aldrich, Arklow, Ireland) in bicarbonate buffer, pH 9.6 with 0.1% BSA. Subsequently, selected wells were coated with 100 nM rmCX_3_CL1 (R&D System, Abingdon, UK) for 1 hr. Since recombinant CX3CL1 is expressed with a C-terminal 6-histidine tag to facilitate purification using nickel agarose, an anti-His tag antibody was coated onto the plate to assist CX3CL1 capture and orientation [25]. CX_3_CR1 cells isolated from bone marrow of CX_3_CR1^gfp/+^ mice were pre-treated with or without CX_3_CR1 peptide antagonist (F1; 2.5 and 5 µM) and were allowed to adhere to wells and then washed with PBS (3×). Attached cells were then labelled with Hoechst dye and counted using UV microscopy. The number of cells per field (10× objective) was expressed as mean ± SEM.

### Mouse model of experimental plaque neovascularization

A murine plaque angiogenesis model previously employed by Sasaki *et al* [26] was used. Mice were anesthetised by intraperitoneal administration of ketamine (90 mg/kg) and xylazine (10 mg/kg) and the left carotid artery at its bifurcation was exposed by standard microsurgical procedure. An 8/0 suture was passed under the artery at its bifurcation and tied to create a complete occlusion of the artery following which the subcutaneous tissue and skin was closed with 6/0 sutures by standard surgical process. The sham mice underwent the surgical steps described above except for the ligation procedure. In treatment groups, two weeks post carotid ligation, the mice were treated with F1 (50 µg total dose) or saline i.p. every third day for 2 weeks. 24 hrs before tissue harvest, the mice were injected with fluorescent microspheres (2–2.5 µm diameter) via tail vein following which the mice were sacrificed by over dose of Sodium Pentobarbital (i.p.).The carotid artery was perfusion fixed using chilled PBS and 4% paraformaldehyde. Perfusion fixed carotid artery was dissected out and cleaned of surrounding fat and cellular debris before being embedded in OCT for cryosectioning.

### In vivo Matrigel neovascularization assay

500 µl of Matrigel (Gibco®; Bio-Sciences, Dublin, Ireland) fortified with 1 ng/ml FGF was subcutaneously implanted into the right and left flank region of mice. At 4 weeks post Matrigel implantation the mice were injected i.v. with 100 µl of 0.05% Evans blue or saline via tail vein and the mice were sacrificed 4 hrs post injection by overdose of sodium pentobarbital (i.p.). In a separate set of studies 200 µg of Rhodamine *Griffonia (Bandeiraea) Simplicifolia* Lectin I(GSL I, BSL I) (Vector Labs, CA, USA) was administered to mice by tail vein 24 hrs before culling. The Evans blue content of Matrigel was determined by dissolving the Matrigel in formic acid and then by reading the absorbance at 620 nm. While the heme content was quantified by pyridine hemochrome spectrophotometric heme assay [27]. Separate Matrigel samples embedded in OCT were processed for immunofluorescence and immunocytochemistry.

### Morphometric and immunofluorescence analysis of injured carotid artery and Matrigel

5 µM thick frozen sections from carotid artery were cut and stained with hematoxylin-Z and eosin and Trichrome stains (CellPath Ltd, Powys, UK). Tissue images were acquired using Nikon CCD camera attached to a Nikon microscope. Morphometric analysis was performed using NIH ImageJ software for data quantification.

For immunofluorescence detection of smooth muscle specific protein and GFP expression carotid artery/Matrigel sections were stained for calponin (rabbit anti calponin: 1∶200; Epitomics, CA, USA), CX_3_CR1 (rabbit anti CX_3_CR1:1∶50; ProSci Incorporated, CA, USA), F4/80 (Rat anti mouse F4/80: 1∶100; eBioscience; Hatfield, UK), Laminin (mouse anti laminin: 1∶500; Novus Biologicals, Cambridge, UK) and/or CD42b (rat anti CD42b: 1∶200; emfret Analytics GmbH & co.KG, Eibelstadt, Germany). Goat anti-rabbit, anti-mouse or anti rat Alexa Fluor 546 or 488 (1∶250 or 500; Abcam, Cambridge, UK) was used as secondary antibody and DAPI was used for nuclear staining. Rabbit, mouse or rat IgG (1∶50 or 200; Abcam, Cambridge, UK) were used as isotype controls. No additional labelling was required for GFP. Several thin optical slices (thickness<0.7 µm) of high-resolution sequential confocal scans (Nikon eC1 plus, TE2000E) of carotid artery sections were acquired to identify single GFP^+^ cells co-expressing smooth muscle marker and to avoid false positive images due to overlapping cells. The acquired images were coded and analysed by two observers blinded to the codes. The 3D reconstruction and analysis of the images was performed using IMARIS software (Bitplane Scientific Software, Zurich, Switzerland).

### *In vitro* cell tubulation and tunneling assay in 3D matrix

CX_3_CR1 positive cells were FACS sorted from whole bone marrow of CX_3_CR1^gfp/+^ and CX_3_CR1^gfp/gfp^ mice and were sandwiched between DQ-red BSA (Invitrogen; Bio-Sciences, Dublin, Ireland) fortified Matrigel layers with CX_3_CL1 gradient in a 15 μ-angiogenesis slide (Ibidi, Munich, Germany). CX_3_CL1 gradient was created by serially layering two layers of Matrigel consisting of 50 and 100 nM rmCX_3_CL1 respectively. The cells were imaged using high-resolution sequential confocal scans (Nikon eC1 plus, TE2000E) at 3 hrs and every day up to day 7 post seeding. A separate series of experiments were conducted in the absence and presence of Rho inhibitor (10 µM; Y27632; MERCK, Darmstadt, Germany). The acquired confocal images were 3D reconstructed and analysed using IMARIS software.

### Extracellular matrix detection

CX_3_CR1 positive cells were FACS sorted from whole bone marrow of CX_3_CR1^gfp/+^ and CX_3_CR1^gfp/gfp^ mice and were grown on 6 well plates at a seeding density of 7×10^5^ cells per well for 5 days with or without 100 nM rmCX_3_CL1 stimulation. In a parallel set of experiments the cell viability was assessed using FACS at day 5 using 7AAD staining. On day 5 the cells were removed by treating with 500 µl of 20 mM Ammonium Hydroxide +0.1% Triton X-100 incubated for 5 mins at 37°C. Following six washes with 1 ml TBS buffer (Tris HCL 50 mM, NaCl 100 mM, pH 8.0), ECM on the plate was removed in the presence of 1% SDS for Western blot analysis. Equal total protein was loaded for Western blot analysis and β-actin was probed as a loading control. The Laminin bands were quantified by digital densitometry normalised to density of respective β-actin bands.

### RhoA activation assay

Active RhoA expression during the in vitro cell tubulation and tunneling process was assessed using a commercial RhoA activation assay kit (Millipore Ireland BV, Cork, Ireland). Briefly, CX_3_CR1 positive cells FACS sorted from whole bone marrow of CX_3_CR1^gfp/+^ mice were plated in 6 well plates at a density of 7×10^5^ cells per well and were stimulated with 100 nM rm CX_3_CL1 for 30 mins. The cells were then lysed (Mg^2+^ lysis/wash buffer) and the lysates were incubated with GST-tagged Rhotekin Rho binding domain for affinity precipitation of GTP-Rho. The bound GTP-Rho protein was pulled-down and resolved on 12% SDS-PAGE. The gel was transferred onto nitrocellulose membrane and detected by immunoblotting using 1∶250 anti RhoA primary antibody (clone 55) and the appropriate peroxidase-conjugated secondary antibody and luminol based chemiluminescent HRP substrate (Millipore Ireland BV, Cork, Ireland). Whole cell lysate samples from all groups were also analysed by SDS-PAGE as total RhoA controls. The active-RhoA bands were quantified by digital densitometry normalised to density of respective total RhoA bands.

### Statistics

All data are represented as mean ± SEM and analysed by unpaired Student t-test or analysis of variance (ANOVA) using Graphpad Prism Version 4 (GraphPad Software, Inc., California, USA). Animal experiments with tissue section analysis were compared based on intra-experiment variation involving 3–4 mice/group that were not littermates.

## Results

### Bone marrow derived CX_3_CR1 positive cells contribute to formation of microvessels

**Figure 1:**
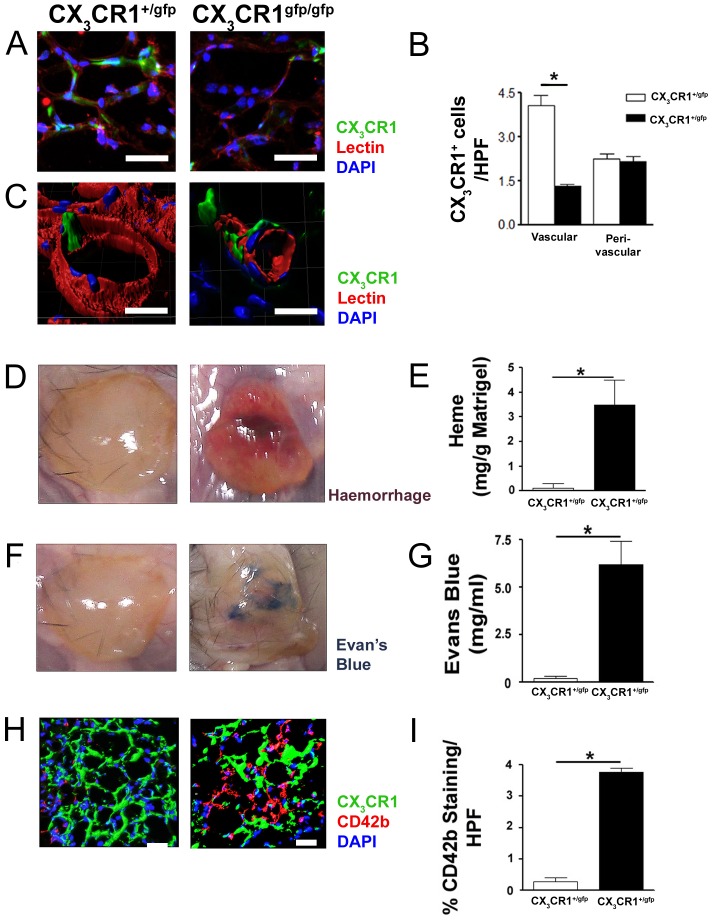
CX_3_CR1 positive cells contribute to formation of microvessels (MV) in Matrigel and deficiency of CX_3_CR1 results in leaky MV phenotype. 500 µl of Matrigel subcutaneously implanted in CX_3_CR1^+/gfp^ and CX_3_CR1^gfp/gfp^ mice for 4 weeks. A, Representative sections of Matrigel isolated from CX_3_CR1^+/gfp^ and CX_3_CR1^gfp/gfp^ mice, intravitally stained with lectin (Rhodamine *Griffonia Simplicifolia*; Red) to show functional MV and DAPI (Nucleus; Blue) show presence of CX_3_CR1 (GFP) positive MV. B, Deficiency of CX_3_CR1 restricted CX_3_CR1 cells to mostly the perivascular region of functional MV within Matrigel. Moreover, the number of integrated vascular associated CX3CR1 positive MV were significantly higher in Matrigels isolated from CX_3_CR1^+/gfp^ mice compared to Matrigels isolated from CX_3_CR1^gfp/gfp^ mice. Data is expressed as mean ± SEM of 25 Matrigel sections/mice (n = 3 independently performed experiments). C, Three dimensional reconstruction of the confocal images using IMARIS indicate CX_3_CR1 positive cells integrated into periluminal vascular wall in the Matrigel implanted in CX_3_CR1^+/gfp^ but not in CX_3_CR1^gfp/gfp^ mice where cells remained in perivascular region (Scale bar: 10 µm). Microvessels formed in Matrigels isolated from CX_3_CR1^gfp/gfp^ mice were leaky, which was evident by increased heme content (D & E), Evan's blue dye leak (F & G), and extravasated platelets (CD42b; Red) (H & I) in the matrix compared to Matrigels implanted in CX_3_CR1^+/gfp^ mice. Data is represented as mean ± SEM of 8 Matrigels/group and 10 Matrigel sections/mice (n = 4 independently performed experiments); * denotes p<0.05.

In this study, myeloid phenotype of CX_3_CR1 expressing cells was established by FACS and tissue immunofluorescence (Figure S1 A, B.) indicating that the CX_3_CR1 transgenic models selected would be suitable for assessing structure function relationships between monocyte/macrophages and evolving neovascularization networks. We first assessed whether CX_3_CR1 positive cells contribute to microvasculature in a well-established *in vivo* model of Matrigel plug neovascularization. Following implantation of Matrigel (500 µl) in functional CX_3_CR1^gfp/+^ and functionally incompetent CX_3_CR1^gfp/gfp^ mice microvascular association (perivascular) and integration(vascular) of CX_3_CR1 positive cells was assessed by immunofluorescence imaging, high resolution confocal microscopy with Z stacking and volume rendering. Functioning microvessels (MV) were identified by intravital staining of rhodamine conjugated lectin injected *via* tail vein. CX_3_CR1 positive cells were noted in the perivascular region of MV and integrated within the walls of MV (Figure 1 A–C). However Matrigels implanted in CX_3_CR1^gfp/+^ mice had significantly increased CX_3_CR1 positive cells integrated into MV wall (4.09±0.34 cells/Matrigel MV/HPF) compared to Matrigels implanted in CX_3_CR1^gfp/gfp^ mice (1.33±0.05 cells/Matrigel MV/HPF) (Figure 1 B, p<0.05) and predominantly a perivascular MV association was observed for CX_3_CR1 positive cells in Matrigel of CX_3_CR1^gfp/gfp^ mice (Figure 1B). Furthermore, we observed increased incidence of leaky vessels in the absence of functional CX_3_CR1, which was evident from increase in Matrigel heme content (Figure 1 D, E, p<0.05), Evans blue dye leak (Figure 1 F, G, p<0.05), and extravasated platelet antigenicity in the extravascular space (Figure 1 H, I, p<0.05) in Matrigel plugs isolated from CX_3_CR1^gfp/gfp^ mice compared to functional CX_3_CR1^gfp/+^ mice.

### Functional CX_3_CR1 positive cells contribute to formation of plaque microvessels

**Figure 2:**
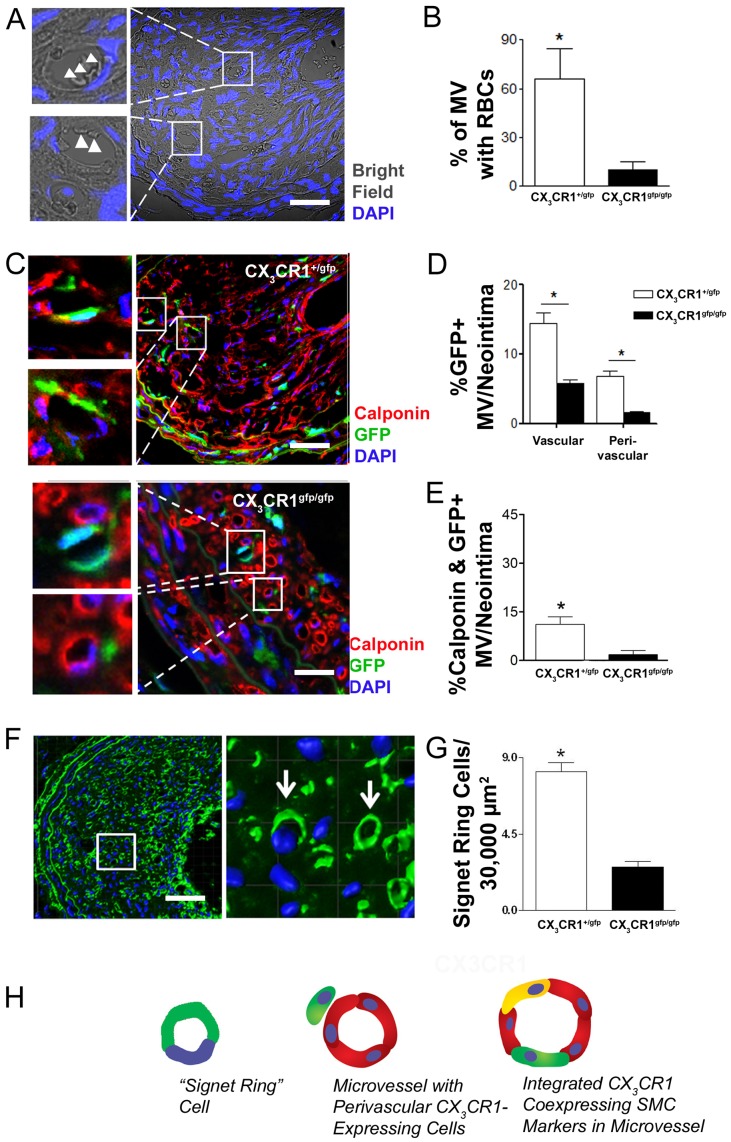
CX_3_CR1 positive cells contribute to formation of experimental plaque angiogenesis. Plaque angiogenesis was created by ligation of carotid artery at its bifurcation for 4 weeks in CX_3_CR1^+/gfp^ and CX_3_CR1^gfp/gfp^ mice. Perfusion fixed carotid arteries were isolated and OCT embedded. 5 µm cross sections were stained with smooth muscle marker (Calponin; Red) and confocal images were acquired. A, Representative bright field cross section image of carotid artery from CX_3_CR1^+/gfp^ mice stained with DAPI (Nucleus; Blue). Red blood cells (RBCs) (arrow heads) were observed in the microvessels indicating these microvessels were functional. B, Mice competent for CX_3_CR1 function had a higher proportion of MV containing RBCs C, Representative cross section image of carotid artery from CX_3_CR1^+/gfp^ and CX_3_CR1^gfp/gfp^ mice were stained with calponin (Red) and DAPI (nucleus; blue). CX_3_CR1 positive cells (GFP positive; Green) integrated into microvascular wall and were also present in perivascular region and co-expressed smooth muscle marker (Calponin; Red) (Scale bar: 10 µm). In the CX_3_CR1 functionally deficient (CX_3_CR1^gfp/gfp^) mice the number of vascular and perivascular cells (GFP positive; Green) (D) and co-expressing smooth muscle marker (E) were significantly reduced. Data is expressed as mean ± SEM of 20 carotid artery cross sections/mice (n = 4 independently performed experiments). F, Microvessels in the plaque stained with CX_3_CR1 (Green) and DAPI (nucleus; blue) were 3D reconstructed using IMARIS software to depict signet ring structures and their tubular architecture. G, The number of signet-ring cells was significantly reduced in CX_3_CR1^gfp/gfp^ mice. H, Schematic representing the three major phenotypes of CX_3_CR1 cells associated with microvascular structures in the plaque. * denotes p<0.05.

Having established the perivascular and intravascular association of CX_3_CR1 cells with angiogenic MV in Matrigel, we investigated whether such an association exists with intimal MV using a recently described carotid artery ligation model of plaque neovascularization in mice [26]. We first established consistent development of carotid artery plaque that was rich in intimal microvessels (MV) with active perfusion as evidenced by presence of red blood cells(RBCs) within MV lumina at 4 weeks post arterial ligation (Figure 2 A). Sham operated animals did not develop plaque neovascularization (data not shown). Significantly greater numbers of intimal MV containing RBCs were noted in CX_3_CR1^gfp/+^ compared to functionally deficient CX_3_CR1^gfp/gfp^ mice (Figure 2 B, p<0.05). Indeed similar to Matrigel neovascularization experiments there was a significantly greater (p<0.05) vascular contribution of CX_3_CR1 positive cells to formation of MV (Figure 2 C, D, E, Figure S2 D, E) in the carotid artery plaque intima in CX_3_CR1^gfp/+^ animals and CX_3_CR1 positive cells were observed in perivascular association with MV within plaque in significantly greater numbers (p<0.05) compared to CX_3_CR1^gfp/gfp^ mice (Figure 2 C, D; Figure S2 D, E). Moreover ∼30% of all MV co- expressed the myeloid marker F4/80 (Figure S2 A, B) and 75% of all GFP positive cells co-expressed F4/80 (Figure S1 B). The total number of GFP positive cells in neointima was similar in CX_3_CR1^gfp/+^ and CX_3_CR1^gfp/gfp^ mice indicating additional chemokine signals to CX3CL1 (Figure S2 C) may be involved in recruitment of monocytes/macrophages to the neointima. Functional deficiency of CX_3_CR1 significantly impaired CX_3_CR1 cell contribution to formation of MV in neointima and in these animals signet-ring like structures were observed. In addition, the total number of signet-ring like cells per unit area were significantly reduced in CX_3_CR1^gfp/gfp^ mice compared to competent CX_3_CR1^gfp/+^ mice (Figure 2 F, G, p<0.05). Of the total MV in the plaque 14.4±1.6% MV were positive for CX_3_CR1 cells associated with MV wall, while 6.8±0.9% MV had perivascularly associated CX_3_CR1 cells (Figure 2 D). However this was significantly (p<0.05) impaired by the functional deficiency of CX_3_CR1 (5.7±0.6% and 1.6±0.1%; vascular wall and perivascular cells respectively). Furthermore 11.1±2.3% MV were positive for CX_3_CR1 cells co-expressing smooth muscle markers (Calponin or α-smooth muscle actin) and this was significantly impaired in the CX_3_CR1^gfp/gfp^ mice (1.8±1.3% MV) (Figure 2 E, Figure S2 D, E, p<0.01). Therefore with respect to intimal microvessel formation in this model CX_3_CR1 positive cells formed either signet ring-like structures, were integrated into MV wall or were perivascularly associated (Figure 2 H) with these MV phenotypes being more mature and more prevalent in the CX_3_CR1^gfp/+^ functionally competent animals.

### Functional deficiency or pharmacological inhibition of CX_3_CR1 results in decreased MV formation and leaky MV phenotype in experimental plaque neovascularization

**Figure 3:**
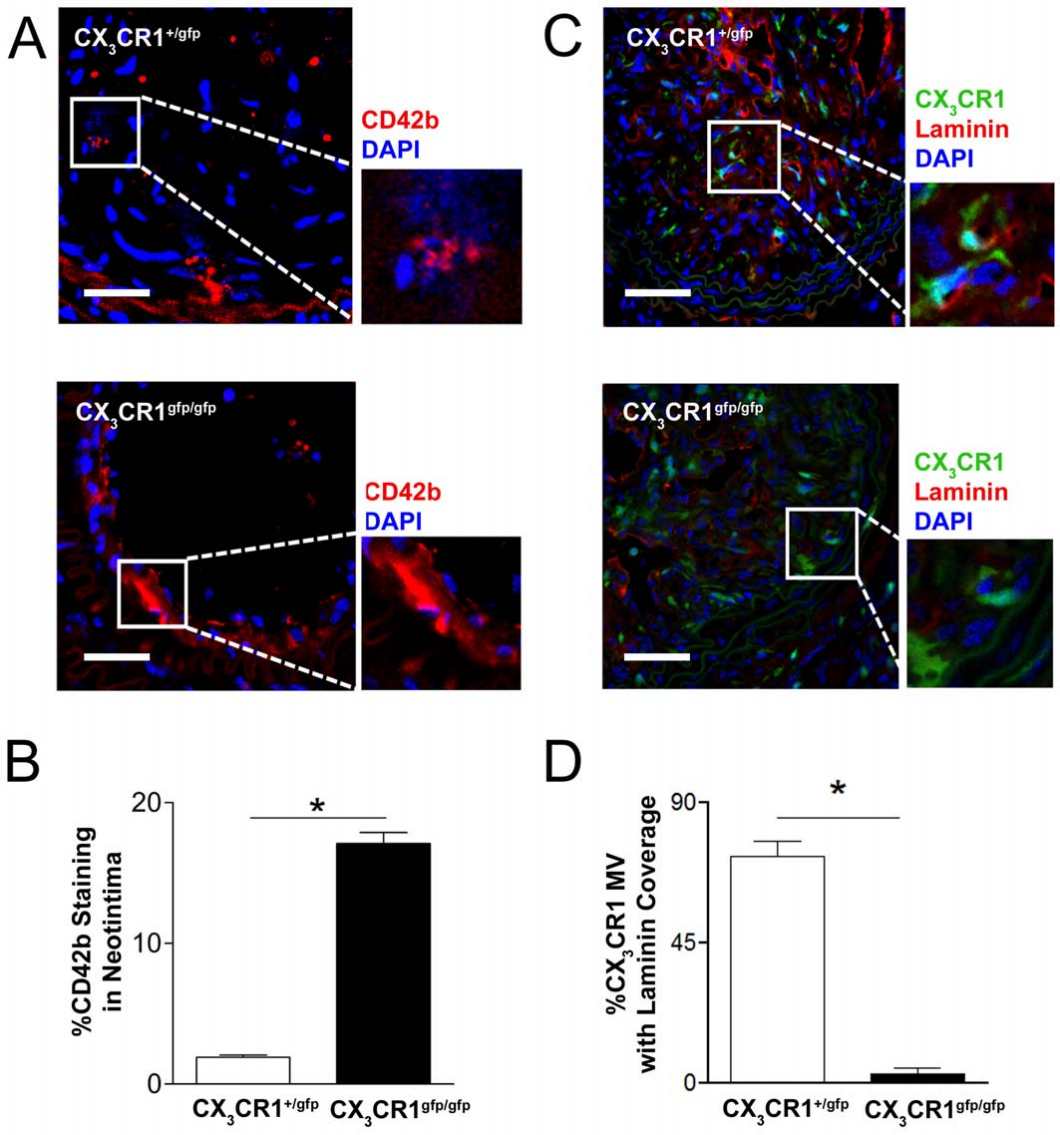
Functional deficiency of CX_3_CR1 results in leaky microvessel phenotype in experimental plaque. Representative cross sectional images of carotid artery plaques from CX_3_CR1^+/gfp^ and CX_3_CR1^gfp/gfp^ mice stained with CD42b (Platelets; Red) (A) or laminin (Basement membrane; Red) (C) and DAPI (Nucleus; Blue). B, Significantly increased staining for platelet CD42b was observed in the neointimal interstitial space in CX_3_CR1^gfp/gfp^ mice compared to competent CX_3_CR1^+/gfp^ mice (Scale bar: 50 µm). D, A significantly greater number of CX_3_CR1 positive microvessels were covered by basement membrane laminin in CX_3_CR1^+/gfp^ mice compared to CX_3_CR1^gfp/gfp^ mice (insets in panel C show GFP and laminin co-staining present in CX_3_CR1^+/gfp^ but not CX_3_CR1^gfp/gfp^ mice). Data is represented as mean ± SEM of 15 plaque sections/mice (n = 4 independently performed experiments); * denotes p<0.05.

In an experimental model of plaque neovascularization, functional deficiency of CX_3_CR1 increased intraplaque haemorrhage (Figure S3 A, B, p<0.01) and enhanced extravasation of platelets in the plaque interstitial space in CX_3_CR1^gfp/gfp^ mice compared to CX_3_CR1 competent animals (Figure 3 A, B, p<0.05), thus indicating a leaky MV phenotype. The leaky MV phenotype in these mice was associated with reduced extracellular matrix and MV basement membrane staining (Figure 3 C, D, p<0.05) in addition to impaired association of CX_3_CR1 cells derived smooth muscle-like cells.

**Figure 4:**
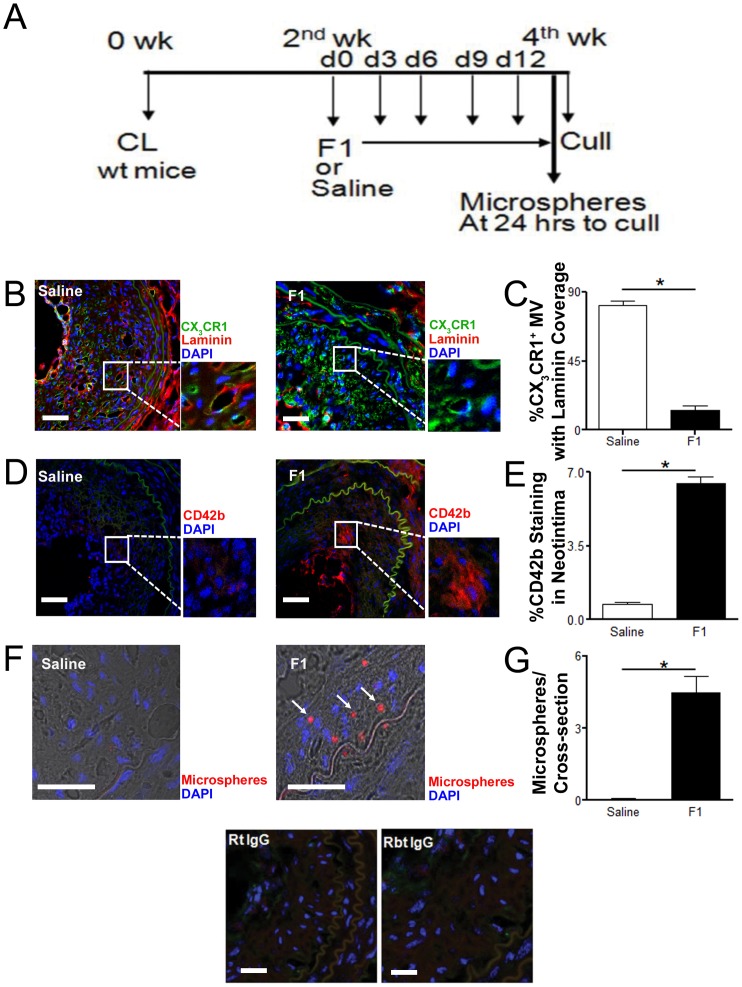
Pharmacologic inhibition of CX_3_CR1 results in formation of leaky microvessels within experimental plaque. A, Drug (F1) study protocol: Carotid artery was ligated in the C57BL6J mice (wt mice) and animals were treated with a selective CX_3_CR1 inhibitor (F1) (from second week post carotid ligation for another two weeks). B, Representative cross sectional images of carotid artery from C57BL6J mice treated with saline or a selective CX_3_CR1 inhibitor (F1) and stained for laminin (B; Basement membrane; Red), or CD42b (D; Platelets; Red), CX_3_CR1 (GFP; Green) and DAPI (Nucleus; Blue). B & C, Significantly greater number of CX_3_CR1 positive microvessels were covered by basement membrane laminin in saline treated C57BL6J mice compared to F1 treated mice. D & E, Increased platelet CD42b staining was observed in the neointimal interstitial space in the F1 treated mice compared to saline treated mice (Scale bar: 50 µm). In addition the leaky microvessel phenotype in mice treated with F1 was confirmed by presence of intravenously administered (tail vein) 2–2.5 µm diameter microspheres (red spheres) in the neointimal lesion (F & G). IgG control staining for isotype-matched antibodies shown. Data is represented as mean ± SEM of 15 plaque sections/mice (n = 4 independently performed experiments); * denotes p<0.01.

To determine whether the effects of genetic functional deletion of CX_3_CR1 receptor on plaque neovascularization could be recapitulated by pharmacological inhibition of receptor activity, we treated wild type C57BL/J6 mice with a CX_3_CL1 analogue (F1 peptide) inhibitor of CX_3_CR1 receptor. The in vivo dose of F1 peptide was optimised based on the capacity of F1 peptide to inhibit binding of CX_3_CR1 positive cells to CX3CL1 coated plates in a static adhesion assay (Figure S4) and on previously reported pharmacodynamic data [24]. 50 µg of F1 peptide was repeatedly administered every third day for 2 weeks by intraperitoneal injection starting from 2 weeks post carotid artery ligation (Figure 4 A). As observed in CX_3_CR1^gfp/gfp^ mice, F1 treated wild type mice exhibited significantly reduced staining for MV associated basement membrane laminin within the plaque (Figure 4 B, C, p<0.01), had reduced extracellular matrix collagen staining (Figure S5 A, B, p<0.01) and had leaky MV phenotype indicated by increased extravasation of platelets (Figure 4 D, E, p<0.01) and intravenously administered microspheres (Figure 4 F, G, p<0.01) in the intimal interstitial space.

### Functional deficiency of CX_3_CR1 impairs 3D Matrigel monocyte/macrophage morphogenesis (tubulation) and tunneling capacity in vitro

**Figure 5:**
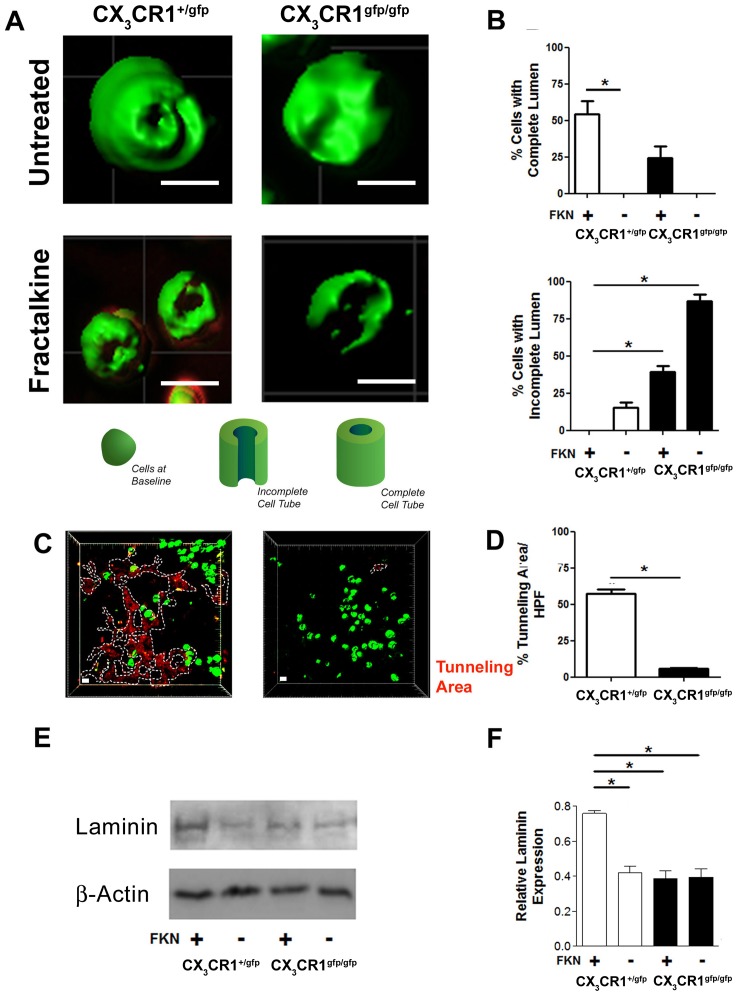
CX_3_CR1 deficient cells lack in vitro tunnelling capacity in solid matrix and form incomplete 3D tube-like structures compared to CX_3_CR1 competent cells. CX_3_CR1 cells isolated from bone marrow of CX_3_CR1^+/gfp^ or CX_3_CR1^gfp/gfp^ mice were sandwiched between two DQ-red fortified (20 µg/ml; substrate for proteases) Matrigel layers with or without 10 ng/ml CX_3_CL1 gradient and were cultured for 5 days. A & B, CX_3_CR1 cells remodelled into tube-like structures (tubulation) especially under CX_3_CL1 gradient (Scale bar: 10 µm). However these tubular structures were incompletely formed by CX_3_CR1 deficient cells. Furthermore, the CX_3_CR1 deficient cells lacked tunneling capacity (dotted lines-protease activation red) in the Matrigel (C & D) and produced significantly less laminin following CX_3_CL1 stimulation (E & F). Data is represented as mean ± SEM of 4 independently performed experiments;* denotes p<0.01.

**Figure 6:**
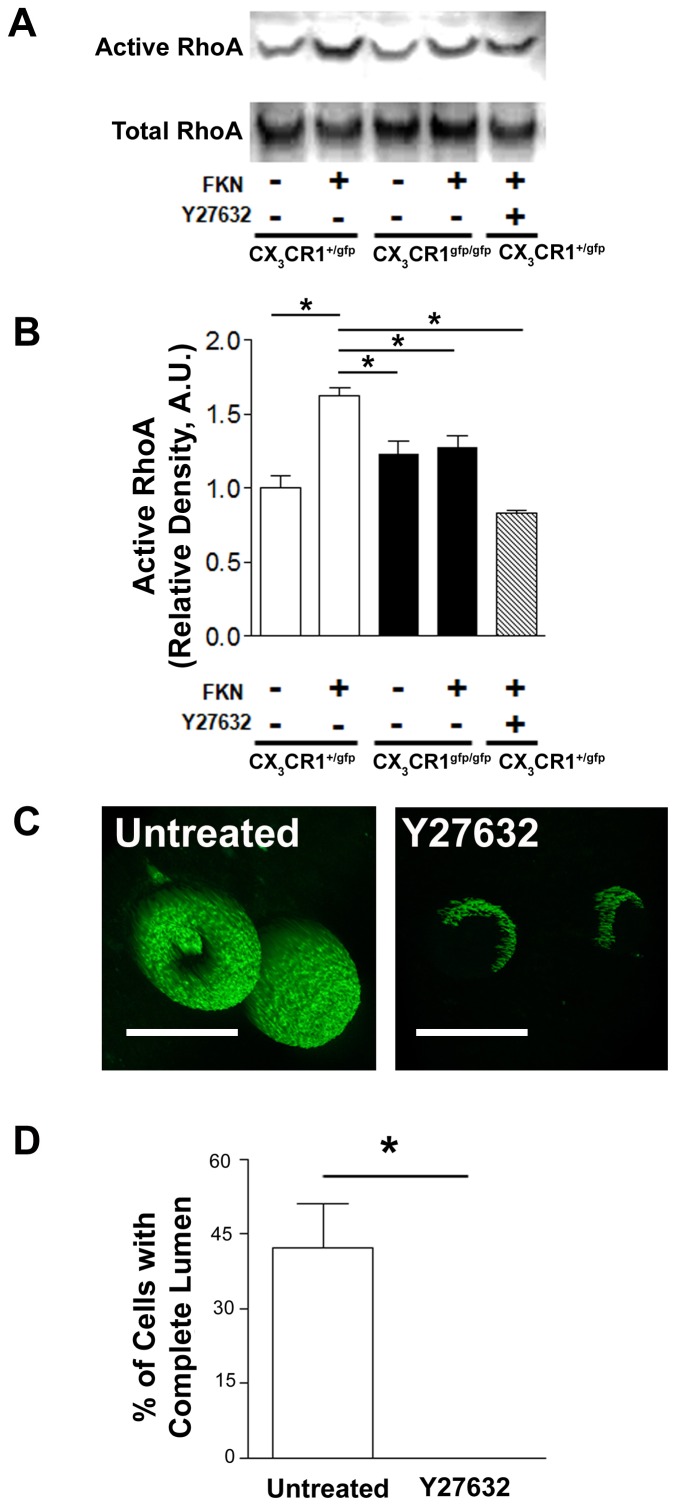
Rho activation is essential for tubulation of CX_3_CL1 stimulated CX_3_CR1 cells. CX_3_CR1 cells isolated from CX_3_CR1^+/gfp^ and CX_3_CR1^gfp/gfp^ mice were treated with 100 nM CX_3_CL1 and 30 minutes post probed for expression of activated RhoA. A, Representative immuno blot showing active RhoA protein expression. B, Active RhoA expression within 30 mins post CX_3_CL1 stimulation was significantly higher in CX_3_CR1 cells isolated from CX_3_CR1^+/gfp^ compared to CX_3_CR1^gfp/gfp^ mice. C, CX_3_CR1 cells isolated from bone marrow of CX_3_CR1^+/gfp^ mice were sandwiched between two Matrigel layers with 10 ng/ml CX_3_CL1 gradient and were cultured for 5 days in absence or presence of Rho inhibitor (Y27632; 10 µM, Scale bar: 10 µm). C & D, Inhibition of Rho using Y27632 (10 µM) prevented the formation of tube-like structures by CX_3_CR1 cells isolated from CX_3_CR1^+/gfp^ mice. Data is represented as mean ± SEM of 4 independently performed experiments;* denotes p<0.01.

To evaluate the morphogenic and cytokinetic effects of CX_3_CR1- CX_3_CL1 interaction cells sandwiched between 2 Matrigel layers were examined in 3D for tube formation in presence or absence of CX_3_CL1 stimulation. CX_3_CR1^gfp/+^ cells formed tube-like structures with clear lumen, an effect augmented by CX_3_CL1 stimulation and in contrast, CX_3_CR1 deficient cells showed impaired tubulation in presence and absence of CX_3_CL1 (Figure 5 A, B, p<0.01). Overall CX_3_CR1 positive cells isolated from both CX_3_CR1^gfp/+^ and CX_3_CR1^gfp/gfp^ mice lacked the ability to form complete tube like structures in the absence of CX_3_CL1 gradient and the number of incompletely tubulated cells was significantly higher under CX_3_CR1 deficient conditions (Figure 5 B, p<0.01). Matrigel tunnelling capacity (Figure 5 C, D) and extracellular matrix (laminin) synthesis (Figure 5 E, F, p<0.01) by CX_3_CR1- CX_3_CL1 stimulated cells isolated from CX_3_CR1^gfp/gfp^ mice was also significantly reduced compared to CX_3_CR1 positive cells isolated from CX_3_CR1^gfp/+^ mice. Importantly equal numbers of cells were viable (44.8–47.2%) in all groups at day 5 in culture. Moreover CX_3_CR1 positive cells from CX_3_CR1^gfp/+^ mice developed larger tubular structures of greater length and with greater external and internal luminal diameter compared to their counterparts from CX_3_CR1^gfp/gfp^ mice (Figure S6 A, B, C, p<0.01). As cellular vacuolation and tubulation have been directly associated with RhoA driven cytoskeletal reorganization [28], [29], [30], we examined active RhoA expression in CX3CL1 stimulated CX_3_CR1 positive cells isolated from CX_3_CR1^gfp/+^ and CX_3_CR1^gfp/gfp^ mice. Initially we observed increased activation of RhoA at 30 minutes post CX3CL1 stimulation of CX_3_CR1 positive cells isolated from CX_3_CR1^gfp/+^ but not CX_3_CR1^gfp/gfp^ mice (Figure 6 A, B, p<0.01). Subsequent inhibition of Rho with an antagonist Y27632 severely impaired RhoA activation (Figure 6 A, B, p<0.01) and the capacity of CX_3_CR1 positive cells to form complete tubular structures in 3D Matrigel sandwich (Figure 6 C, D, p<0.01).

## Discussion

The major and new finding of this study is that functional competence of the monocyte/macrophage chemokine receptor CX_3_CR1 is essential for nascent microvessel formation, structural integrity and maturation in two models of neovascularization. Moreover, monocyte/macrophage tubulation, extracellular matrix tunneling and differentiation into smooth muscle-like cells within the microvasculature *in vivo* is driven in part by CX_3_CR1 interaction with its cognate ligand. CX_3_CR1-CX_3_CL1 association activates RhoA signalling, a central pathway in neovascularization/angiogenesis initiation and maintenance [29]. Abrogation of CX_3_CL1-CX_3_CR1 interaction reduces mural cell investment of nascent proangiogenic tubes in both models of neovascularization and reduces extracellular matrix protein production of laminin and collagen in a plaque neovascularization model (Figure S5). Finally loss of CX_3_CR1 function or therapeutic targeting of CX_3_CR1 interaction using a mutant peptide CX_3_CL1-mimetic leads to development of smaller, poorly developed, leaky and haemorrhagic microvessels in Matrigel neovascularization and experimental plaque neovascularization models respectively.

Monocyte/macrophage regulation of neovascularization has been extensively investigated over the past decade and is known to involve both paracrine [31] and cell-cell interactions [32]. Moreover, pioneering studies by Moldovan and colleagues have demonstrated that monocyte-macrophages drill tunnels in extracellular matrix to facilitate initial ingrowth of new capillaries [4], [12], [13], [33]. Thus monocyte/macrophages can provide paracrine and scaffold support for initiation, expansion and maturation of microvascular networks. However little is known about which myeloid cell population or surface receptors are implicated in signalling and morphogenesis and maturation of neovascular networks.

In both models of neovascularization used in our study it is likely that CX_3_CL1 [28] as well as other chemokines [12], [13] initiated recruitment of CX_3_CR1 positive cells into areas of microvessel growth. Although CX_3_CR1 is the only known receptor for CX_3_CL1, a recent study reported CX_3_CL1 induced angiogenesis, cell tubulation and signalling being partially insensitive to pertussis toxin pre-treatment [21]. Interestingly we also observed CX_3_CL1 induced tubulation events in CX_3_CR1 deficient cells (Figure 5B) and signet ring like structures in carotid artery plaques from CX_3_CR1 deficient mice (Figure 2C), thus indicating possibility of CX_3_CR1 independent effects of CX_3_CL1. Nevertheless, we show here that for the most part competence of CX_3_CR1 cells to interact with CX_3_CL1 ligand likely determines the spectrum of cell differentiation events that subsequently occurs post chemokine-receptor interaction [17], [34], [35]. Previously, we have identified smooth muscle cell differentiation as an important effect of CX_3_CL1-CX_3_CR1 interaction post arterial wire injury [17]. In the current study we extend these findings to a spectrum of additional downstream effects starting with augmented RhoA signalling, a pathway central to neovascularization/angiogenesis initiation with key functions regulating actin cytoskeleton, cell polarity and vacuolation [29]. We show *in vitro* that CX_3_CR1- CX_3_CL1 interaction increases RhoA activation and that monocyte/macrophage vacuolation downstream of CX_3_CL1 activation is completely abrogated by a Rho antagonist-Y27632 suggesting this cytoskeletal effect of CX_3_CR1 receptor activation may be driven at least in part by a Rho dependent mechanism. Since Rho activation initiates multiple other downstream pathways including smooth muscle cell differentiation [36] and extracellular matrix production [37], [38], [39], [40], [41] it is not surprising that CX_3_CR1– CX_3_CL1 interaction also augments laminin production and extracellular matrix tunnelling and migration activity of these activated monocyte/macrophages.

Consistent with these *in vitro* findings we show that CX_3_CR1 positive cells also exhibit a distinct tubulation phenotype change *in vivo* in the form of signet ring cell (a single DAPI nucleus and GFP positive ring cytoplasm) formation (Figure 2F). Monocyte/macrophage tubulation (signet-ring formation) was impaired in CX_3_CR1 deficient mice and together with previous work by Moldovan and colleagues [4], [13] it is conceivable that impairment of Rho dependent cytoskeletal changes may have impaired initiation and maturation of nascent microvessels in these animals. Although no RBCs were observed within these tubular single cell structures we could readily demonstrate RBCs within two other MV-associated CX_3_CR1 phenotypes namely perivascular and mural (vascular) cell types. Consistent with our previous evidence for CX_3_CR1 differentiation into SM-like cells we show that almost all mural CX_3_CR1 positive cells within MVs co-express smooth muscle markers [16], [17]. More importantly functional deficiency of CX_3_CR1 markedly reduced *in vivo* mural and perivascular association of CX_3_CR1 cells within plaque MVs and mural association within Matrigel MV. Functional deficiency of CX_3_CR1 also reduced MV associated extracellular matrix production with more than 70% reduction in MV-associated laminin and collagen staining in CX_3_CR1 deficient mice. The functional consequences of aberrant CX_3_CR1 phenotype included increased microvascular leakage of blood, platelets, and intravascularly administered solutes (Evans Blue dye). It is tempting to speculate whether failure of CX_3_CR1 deficient cells to tubulate, or initiate differentiation into mural smooth muscle-like cells or impairment of extracellular matrix production may be significant contributors to increased microvessel porosity to blood and solutes *in vivo* thus providing a mechanism underlying the leaky MV phenotype. It is also conceivable that reduced mural CX_3_CR1 positive cells may have effects on microvascular endothelial permeability [42].

Our current findings contribute to emerging literature on the direct involvement of monocyte/macrophages to smooth muscle cell differentiation [35], [43], [44] and extra cellular matrix [43], [44], [45] production within the vessel wall and supports the hypothesis of CX_3_CR1 positive cells contributing to formation of stable MV within neovascular networks and within experimental plaque. The monocyte/macrophage contribution to stable MV may thus occur via a number of mechanisms including inter alia differentiation of myeloid cells into smooth muscle cells, intra-mural and perivascular cell integration, synthesis of basement membrane, and paracrine secretion of other extracellular matrix support factors by these cells. Additionally CX_3_CR1 positive cells contribute to formation of stable MV in a plaque-independent setting, as indicated by our *in vivo* Matrigel induced neovascularization data.

While the implantation of a subcutaneous Matrigel plug is accepted as an excellent model of *in vivo* neovascularization, controversy remains on the appropriateness of current plaque neovascularization models used in mice. This limitation was recently addressed by Sasaki *et al* [46] wherein ligation of common carotid artery in a murine model was shown to develop a lesion with reproducible intimal neovascularization [46]. Thus we adapted this model for our current CX_3_CR1 studies and provide clear evidence of functional MV formation in this model using high resolution 3D confocal imaging.

Given that microvessel integrity and function is increasingly investigated as a potential contributor to plaque growth and stability we examined whether therapeutic targeting of CX_3_CR1 would alter MV structure and function. Remarkably, treatment of wild type mice with a CX_3_CL1 peptide-mimetic (F1) that specifically antagonized CX_3_CR1 recapitulated the leaky microvessel phenotype seen in CX_3_CR1 deficient mice. These F1 treated animals also exhibited marked reduction in basement membrane-laminin MV coverage, increased platelet extravasation and excess leakage of intravascularly administered microspheres compared to control treated animals. These data confirm that CX_3_CR1 deficiency or specific small molecule targeting of CX_3_CR1 reduces neovascularization (Figure S7, S8) and alters microvascular structure *in vivo*. Our data also illustrate that therapeutic interference in microvascular structure-function may potentially be a double edged sword with unintended consequences such as microvascular leakage. This is especially relevant given the recent increase in cardiovascular events observed in patients receiving anti-angiogenic therapy for cancer (VEGF inhibitor-Avastin) [47]. Thus, although enthusiasm is growing for targeting plaque neovascularization in humans our data suggests caution is required as complex interactions between evolving microvasculature and plaque growth and stability may not be easily amenable to single molecule therapeutic approaches.

## Supporting Information

Figure S1 Myeloid phenotype of CX_3_CR1-GFP cells. A, Flow cytometric analysis of the CD45/CX_3_CR1-GFP dual positive cell population of mouse bone marrow cells showing ∼85% or greater coexpression of GFP cells with cfms, CD11b or F4/80, with lower levels of coexpression with Gr-1. Data is representative of 3 independently performed experiments, the values inset are mean ± SEM of 3 independently performed experiments. B, CX_3_CR1-GFP and F4/80 co-staining in plaque (upper panels) and Matrigel angiogenesis (lower panels) models showing majority of CX_3_CR1-GFP cells positive for myeloid marker (F4/80). Data is expressed as mean ± SEM of 10 carotid artery/Matrigel cross sections/mice (n = 3 independently performed experiments).
(TIF)

Figure S2 Contribution of myeloid cells to formation of microvessels in neointima. A, Representative cross section image of carotid artery from wild type mice stained with myeloid (F4/80) and smooth muscle (calponin) marker. Nuclei are stained with DAPI (Blue). B, ∼30% of microvessels in the neointima were positive for myeloid marker and ∼15% of microvessels co-expressed myeloid and smooth muscle marker. C, The total number of GFP positive cells in the neointima was similar in CX_3_CR1^+/gfp^ and CX_3_CR1^gfp/gfp^ mice. Data is expressed as mean ± SEM of 20 carotid artery cross sections/mice (n = 3 animals). D, Representative cross section image of carotid artery from CX_3_CR1^+/gfp^ and CX_3_CR1^gfp/gfp^ mice were stained with α-smooth muscle actin (α-SMA; Red) and DAPI (nucleus; blue). CX_3_CR1 positive cells (GFP positive; Green) integrated into microvascular wall and were also present in perivascular region and co-expressed smooth muscle marker (α-SMA; Red) (Scale bar: 10 µm). E, In the CX_3_CR1 functionally deficient (CX_3_CR1^gfp/gfp^) mice the number microvascular CX_3_CR1 positive cells co-expressing smooth muscle marker were significantly reduced. Data is expressed as mean ± SEM of 20 carotid artery cross sections/mice (n = 4 independently performed experiments). * denotes p<0.01.
(TIF)

Figure S3 Neointimal lesions from CX_3_CR1^gfp/gfp^ mice show increased intra plaque haemorrhage. A, Representative cross section image of carotid artery from CX_3_CR1^+/gfp^ and CX_3_CR1^gfp/gfp^ mice stained with Hematoxylin and Eosin, showing increased intraplaque haemorrhage (Dark red staining). B, Data is expressed as mean ± SEM of 20 carotid artery cross sections/mice (n = 4 independently performed experiments); * denotes p<0.01.
(TIF)

Figure S4 Dose dependent inhibition of CX_3_CR1 cells to CX_3_CL1 coated plates by selective CX_3_CR1 inhibitor (F1) in a static cell adhesion assay. Data is represented as mean ± SEM of 4 independently performed experiments; * denotes p<0.01.
(TIF)

Figure S5 A, Pharmacological inhibition of CX_3_CR1 receptors using CX_3_CR1 inhibitor (F1) reduces collagen staining (light blue on trichrome) in the neointima. B, Data is expressed as mean ± SEM of 20 carotid artery cross sections/mice (n = 4 independently performed experiments); * denotes p<0.01.
(TIF)

Figure S6 Cell length (A), cell diameter (B) and lumen diameter (C) of CX3CR1 cells isolated from CX_3_CR1^+/gfp^ or CX_3_CR1^gfp/gfp^ mice cultured for 5 days in Matrigel sandwich with 10 ng/ml CX_3_CL1 gradient. Data is represented as mean ± SEM of 4 independently performed experiments;* denotes p<0.01.
(TIF)

Figure S7 Neointimal lesions from CX_3_CR1^gfp/gfp^ mice are significantly reduced compared to CX_3_CR1^+/gfp^ mice. Data is expressed as mean ± SEM of 20 carotid artery cross sections/mice (n = 8 independently performed experiments); * denotes p<0.01.
(TIF)

Figure S8 Pharmacological inhibition of CX_3_CR1 receptors using CX_3_CR1 inhibitor (F1) significantly reduces the neointimal area. Data is expressed as mean ± SEM of 15 carotid artery cross sections/mice (n = 4 independently performed experiments); * denotes p<0.01.
(TIF)
