# Supplementary figures and images for: Role of CX3CR1 Receptor in Monocyte/Macrophage Driven Neovascularization
Source: PLoS One. 2013 Feb 21;8(2):e57230. doi: 10.1371/journal.pone.0057230 (PMC3578809; doi:10.1371/journal.pone.0057230)

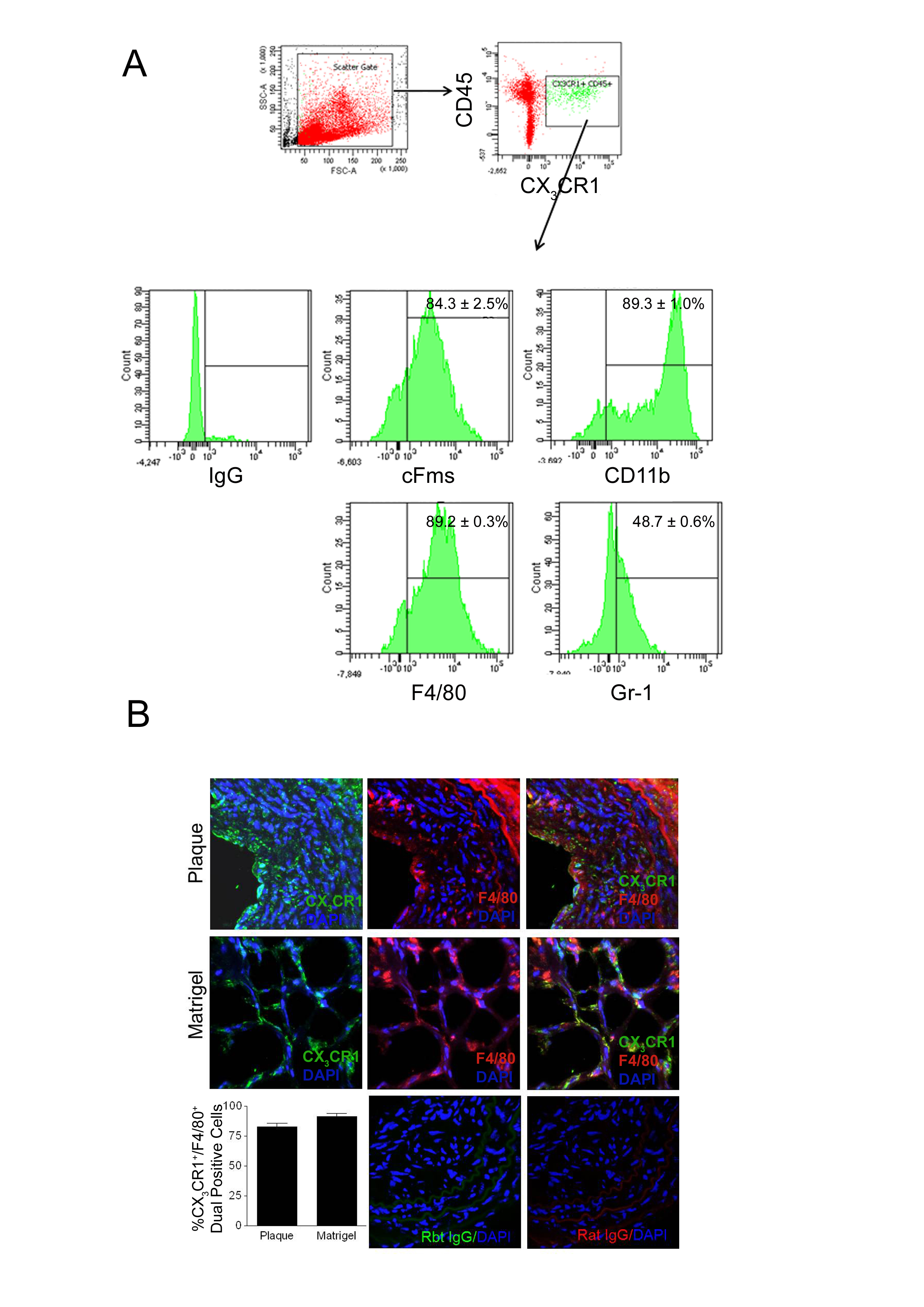

Supplement: Figure S1 — Myeloid phenotype of CX3CR1-GFP cells. A, Flow cytometric analysis of the CD45/CX3CR1-GFP dual positive cell population of mouse bone marrow cells showing ∼85% or greater coexpression of GFP cells with cfms, CD11b or F4/80, with lower levels of coexpression with Gr-1. Data is representative of 3 independently performed experiments, the values inset are mean ± SEM of 3 independently performed experiments. B, CX3CR1-GFP and F4/80 co-staining in plaque (upper panels) and Matrigel angiogenesis (lower panels) models showing majority of CX3CR1-GFP cells positive for myeloid marker (F4/80). Data is expressed as mean ± SEM of 10 carotid artery/Matrigel cross sections/mice (n = 3 independently performed experiments). (TIF) [file pone.0057230.s001.tif]

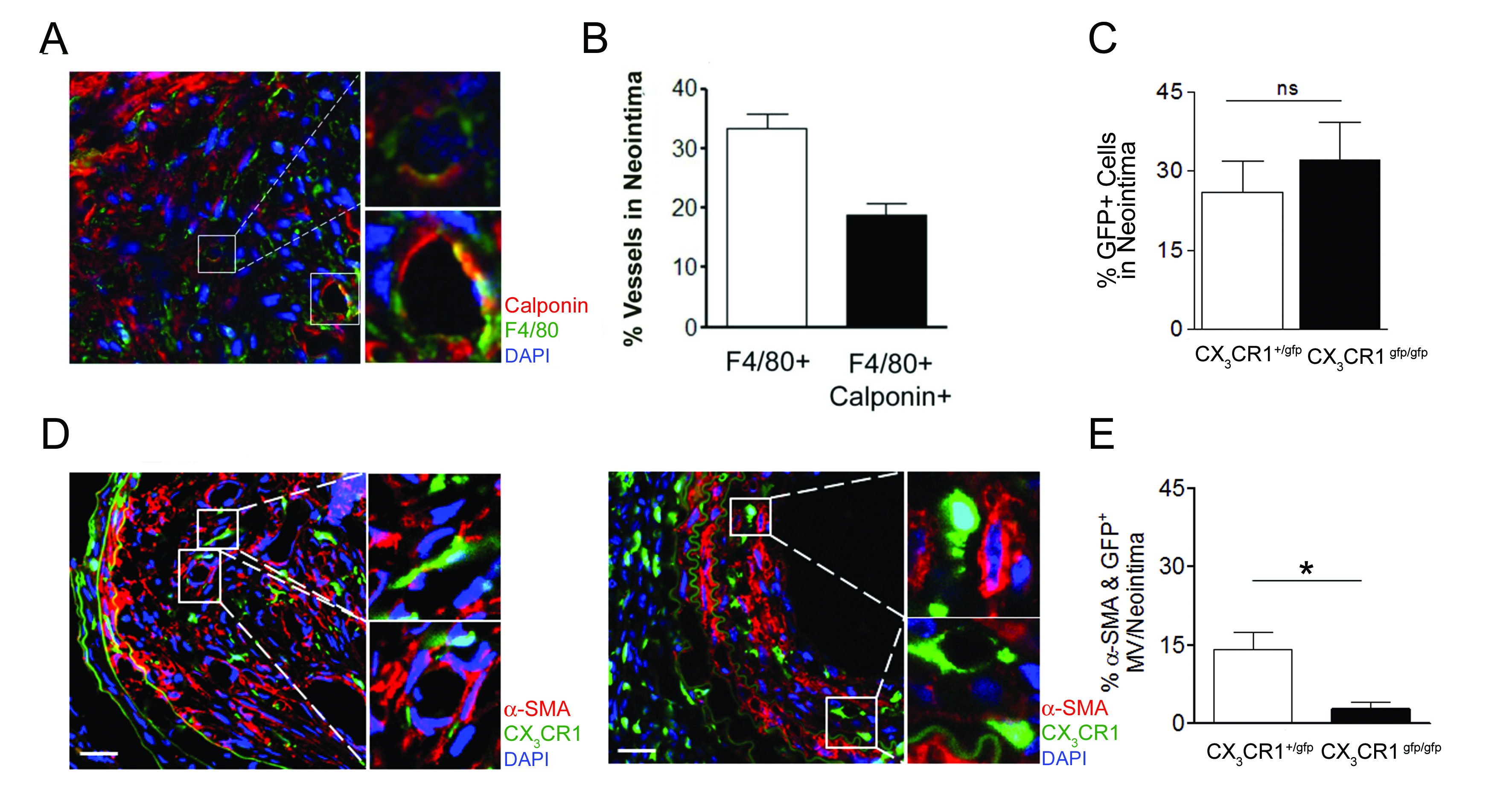

Supplement: Figure S2 — Contribution of myeloid cells to formation of microvessels in neointima. A, Representative cross section image of carotid artery from wild type mice stained with myeloid (F4/80) and smooth muscle (calponin) marker. Nuclei are stained with DAPI (Blue). B, ∼30% of microvessels in the neointima were positive for myeloid marker and ∼15% of microvessels co-expressed myeloid and smooth muscle marker. C, The total number of GFP positive cells in the neointima was similar in CX3CR1+/gfp and CX3CR1gfp/gfp mice. Data is expressed as mean ± SEM of 20 carotid artery cross sections/mice (n = 3 animals). D, Representative cross section image of carotid artery from CX3CR1+/gfp and CX3CR1gfp/gfp mice were stained with α-smooth muscle actin (α-SMA; Red) and DAPI (nucleus; blue). CX3CR1 positive cells (GFP positive; Green) integrated into microvascular wall and were also present in perivascular region and co-expressed smooth muscle marker (α-SMA; Red) (Scale bar: 10 µm). E, In the CX3CR1 functionally deficient (CX3CR1gfp/gfp) mice the number microvascular CX3CR1 positive cells co-expressing smooth muscle marker were significantly reduced. Data is expressed as mean ± SEM of 20 carotid artery cross sections/mice (n = 4 independently performed experiments). * denotes p<0.01. (TIF) [file pone.0057230.s002.tif]

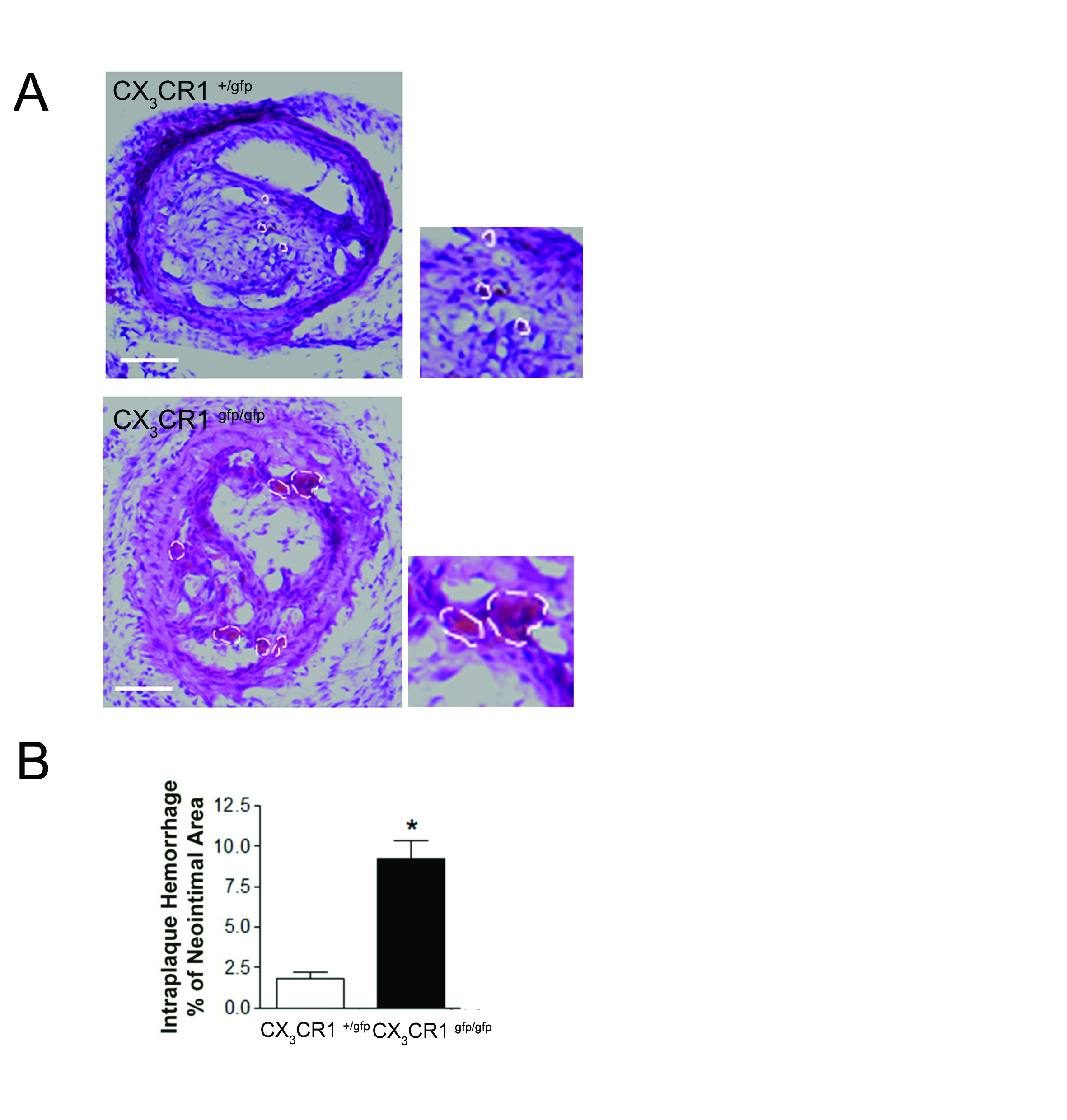

Supplement: Figure S3 — Neointimal lesions from CX3CR1gfp/gfp mice show increased intra plaque haemorrhage. A, Representative cross section image of carotid artery from CX3CR1+/gfp and CX3CR1gfp/gfp mice stained with Hematoxylin and Eosin, showing increased intraplaque haemorrhage (Dark red staining). B, Data is expressed as mean ± SEM of 20 carotid artery cross sections/mice (n = 4 independently performed experiments); * denotes p<0.01. (TIF) [file pone.0057230.s003.tif]

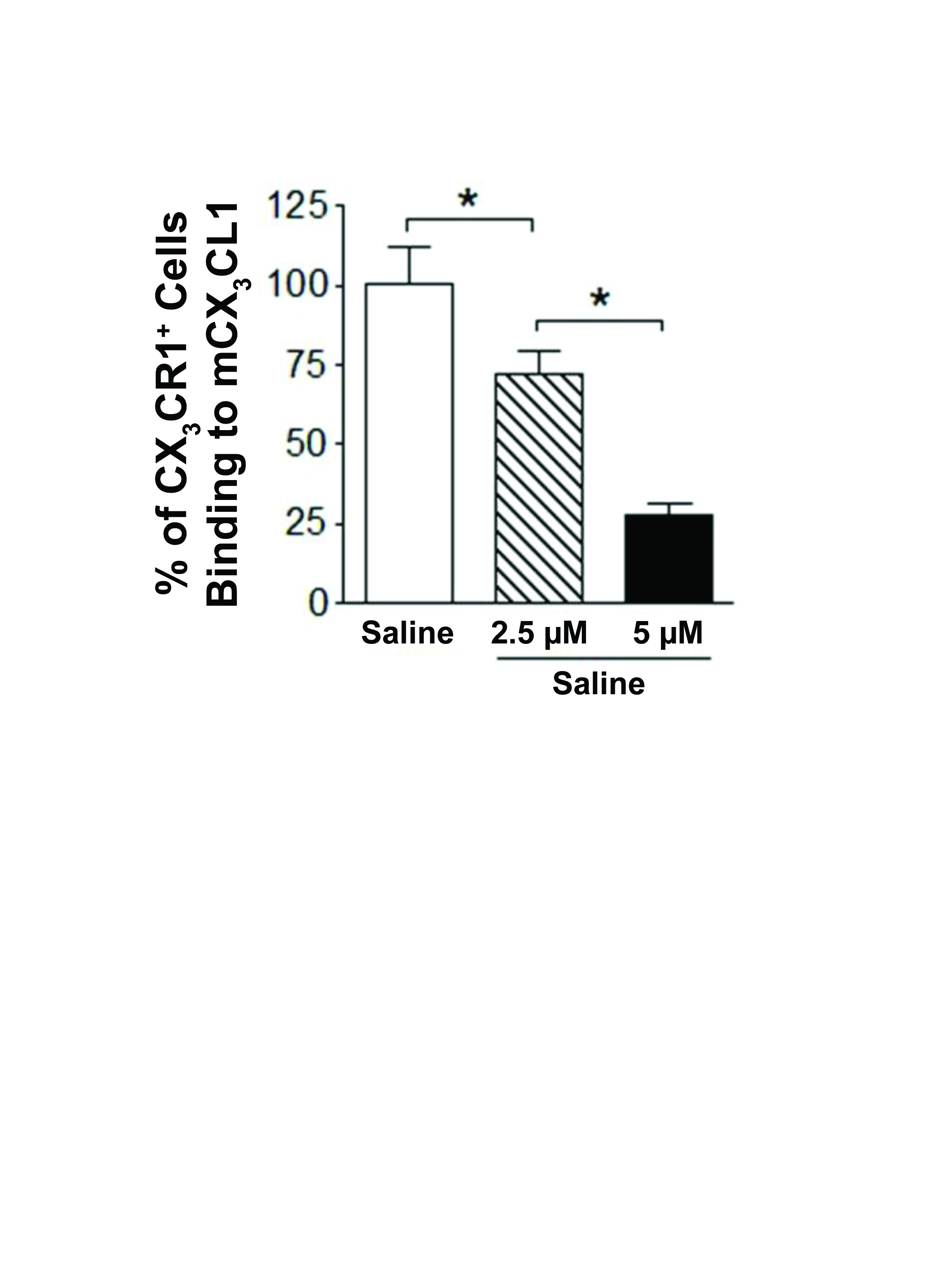

Supplement: Figure S4 — Dose dependent inhibition of CX3CR1 cells to CX3CL1 coated plates by selective CX3CR1 inhibitor (F1) in a static cell adhesion assay. Data is represented as mean ± SEM of 4 independently performed experiments; * denotes p<0.01. (TIF) [file pone.0057230.s004.tif]

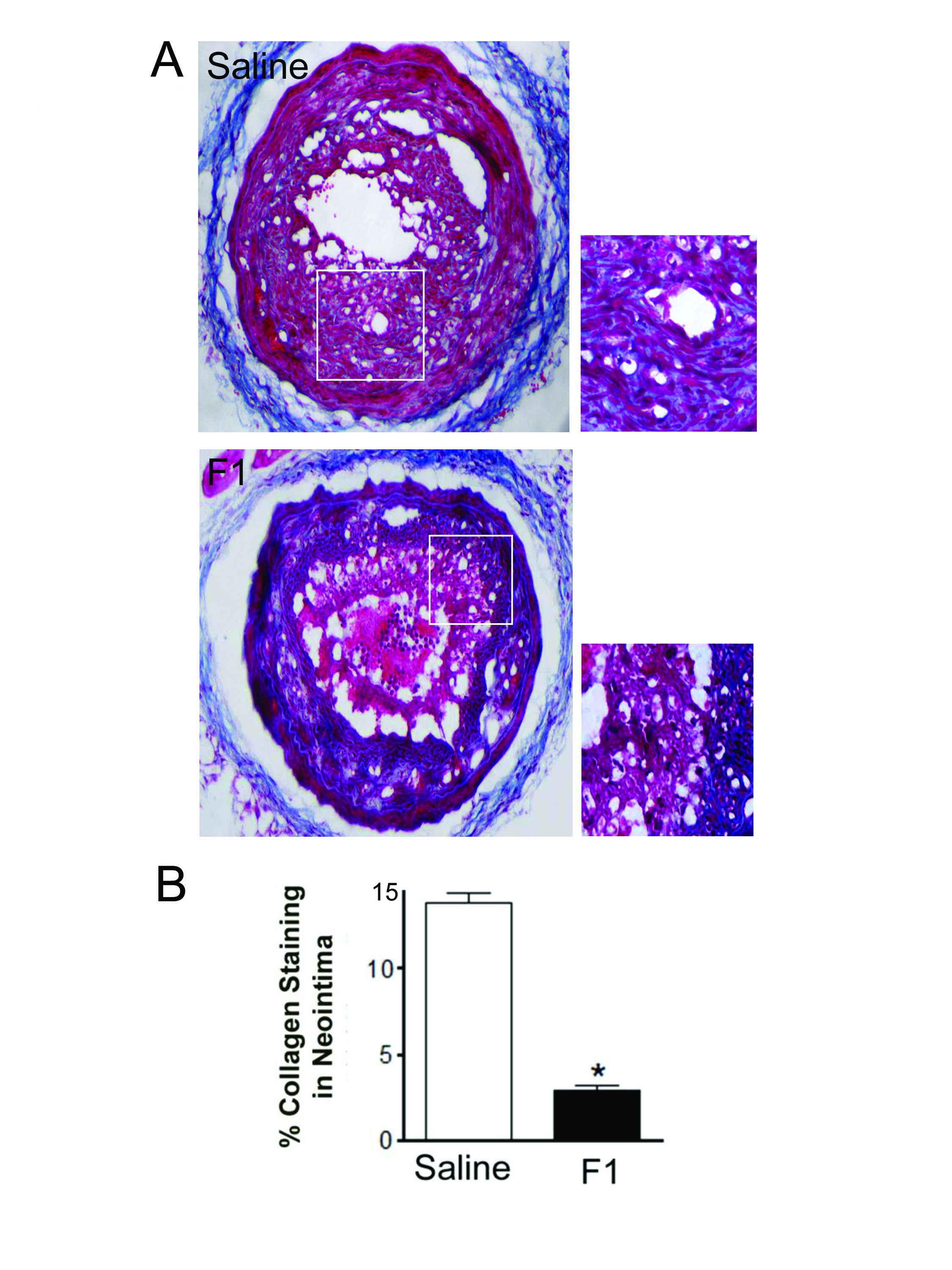

Supplement: Figure S5 — A, Pharmacological inhibition of CX3CR1 receptors using CX3CR1 inhibitor (F1) reduces collagen staining (light blue on trichrome) in the neointima. B, Data is expressed as mean ± SEM of 20 carotid artery cross sections/mice (n = 4 independently performed experiments); * denotes p<0.01. (TIF) [file pone.0057230.s005.tif]

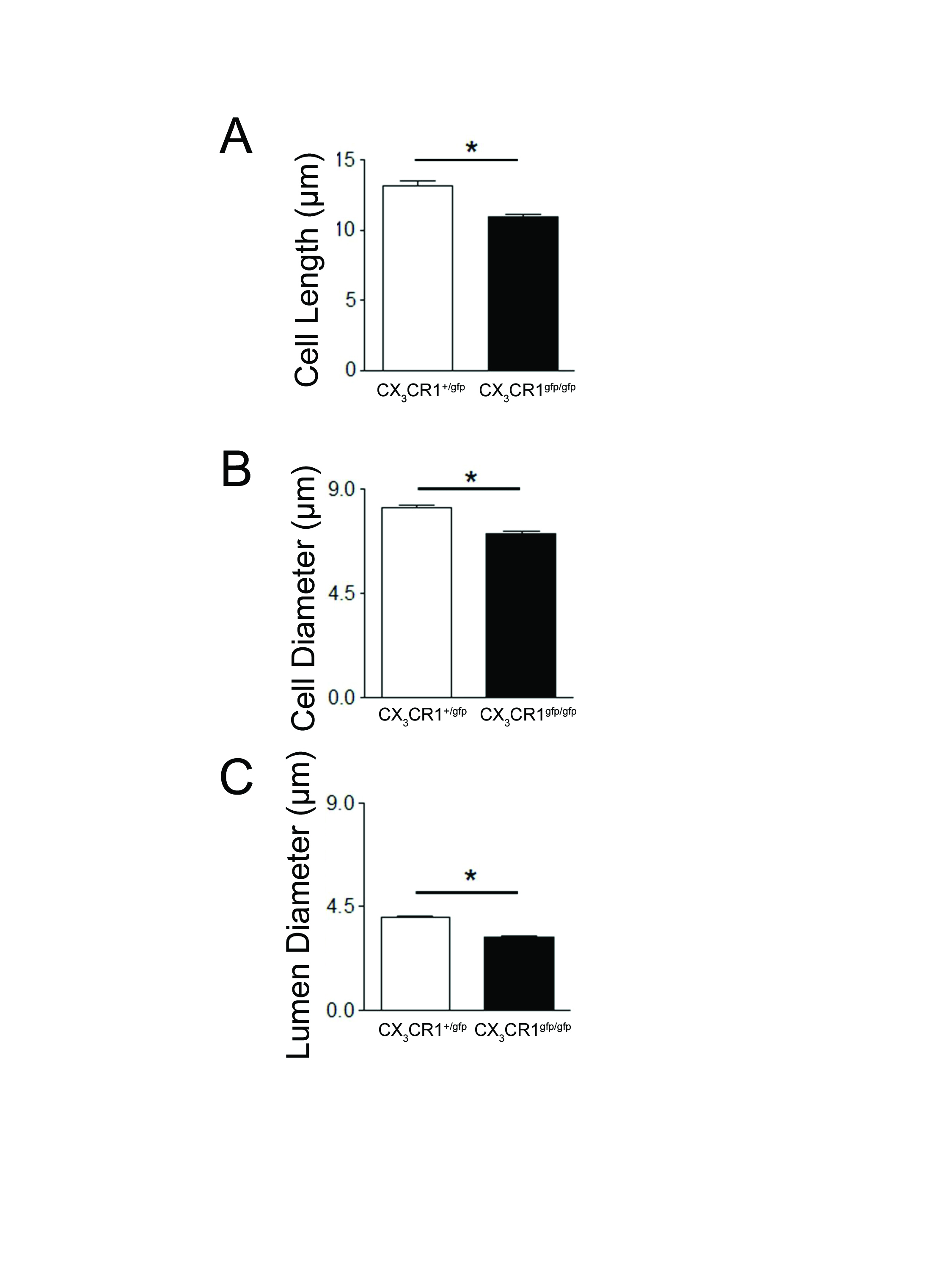

Supplement: Figure S6 — Cell length (A), cell diameter (B) and lumen diameter (C) of CX3CR1 cells isolated from CX3CR1+/gfp or CX3CR1gfp/gfp mice cultured for 5 days in Matrigel sandwich with 10 ng/ml CX3CL1 gradient. Data is represented as mean ± SEM of 4 independently performed experiments;* denotes p<0.01. (TIF) [file pone.0057230.s006.tif]

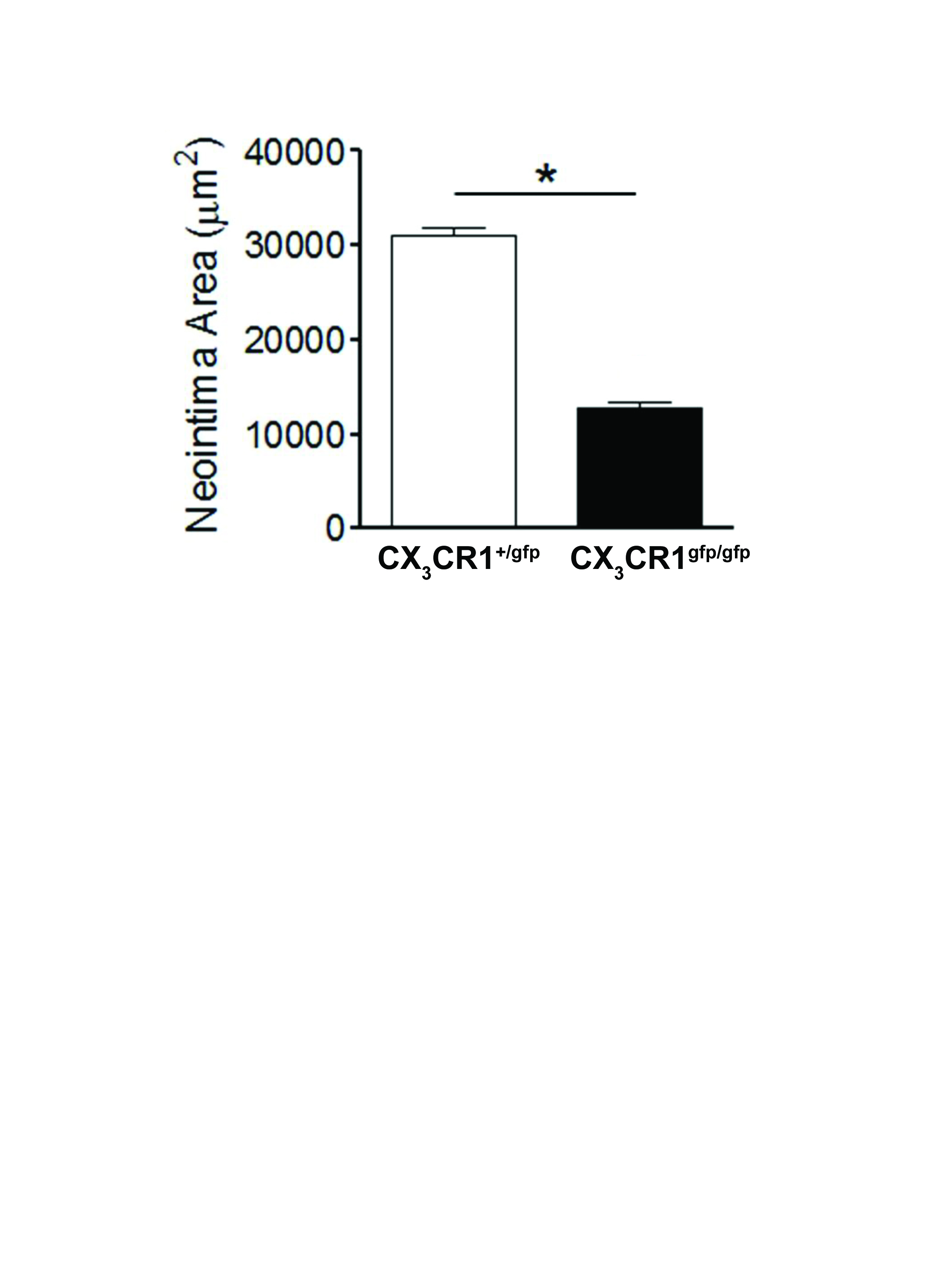

Supplement: Figure S7 — Neointimal lesions from CX3CR1gfp/gfp mice are significantly reduced compared to CX3CR1+/gfp mice. Data is expressed as mean ± SEM of 20 carotid artery cross sections/mice (n = 8 independently performed experiments); * denotes p<0.01. (TIF) [file pone.0057230.s007.tif]

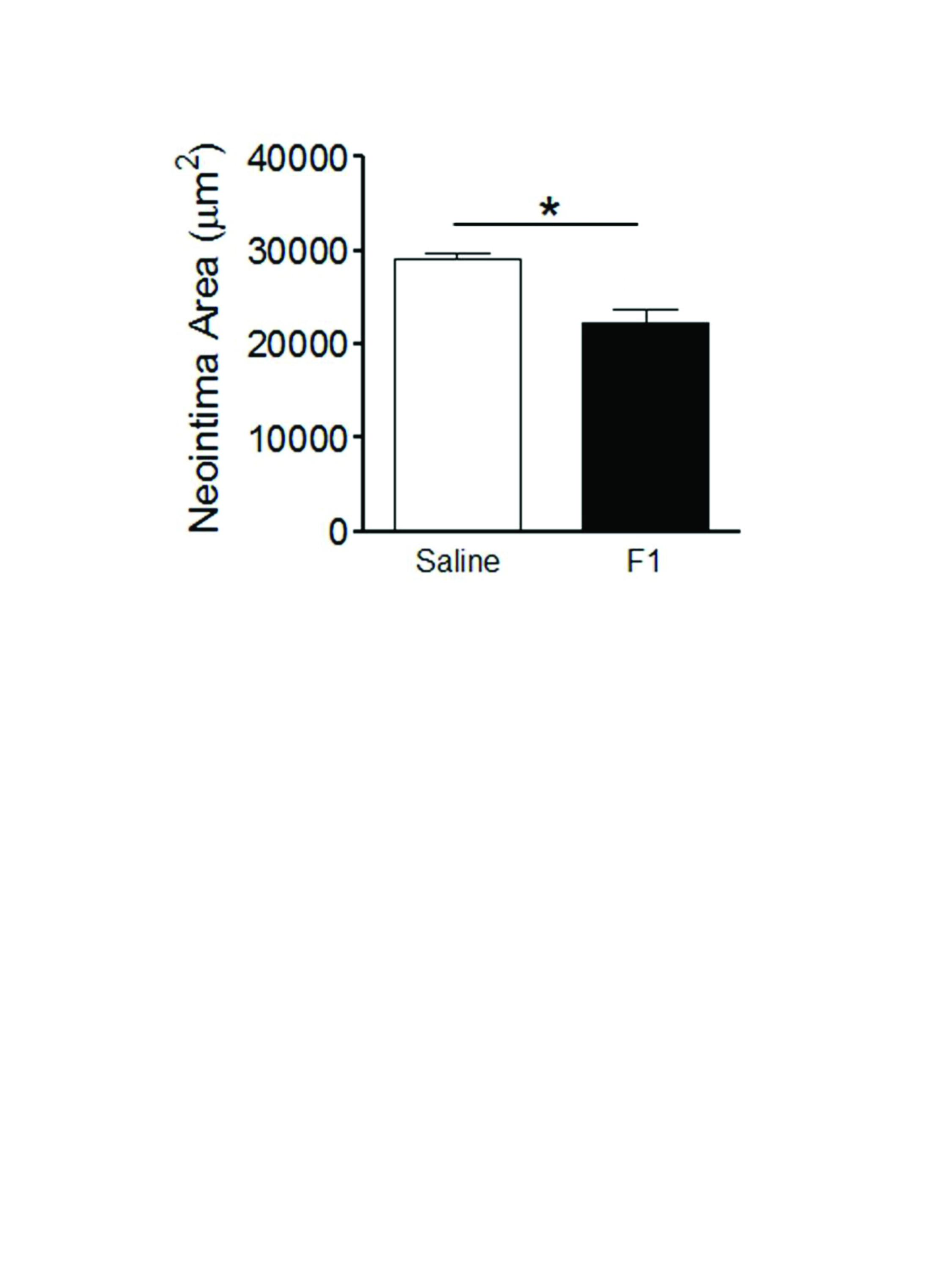

Supplement: Figure S8 — Pharmacological inhibition of CX3CR1 receptors using CX3CR1 inhibitor (F1) significantly reduces the neointimal area. Data is expressed as mean ± SEM of 15 carotid artery cross sections/mice (n = 4 independently performed experiments); * denotes p<0.01. (TIF) [file pone.0057230.s008.tif]
